# Tiny clue reveals the general trend: a bibliometric and visualized analysis of renal microcirculation

**DOI:** 10.1080/0886022X.2024.2329249

**Published:** 2024-03-14

**Authors:** Bing Wang, Mengting Xu, Sunjing Fu, Yingyu Wang, Hao Ling, Yuan Li, Bingwei Li, Xueting Liu, Qin Ouyang, Xiaoyan Zhang, Ailing Li, Xu Zhang, Mingming Liu

**Affiliations:** a Institute of Microcirculation, Chinese Academy of Medical Sciences and Peking Union Medical College, Beijing, China; b International Center of Microvascular Medicine, Chinese Academy of Medical Sciences, Beijing, China; c Department of Radiology, The Affiliated Changsha Central Hospital, Hengyang Medical School, University of South China, Changsha, China; d Department of Pathology, Wangjing Hospital, China Academy of Chinese Medical Science, Beijing, China; e Laboratory of Electron Microscopy, Ultrastructural Pathology Center, Peking University First Hospital, Beijing, China; f Diabetes Research Center, Chinese Academy of Medical Sciences & Peking Union Medical College, Beijing, China

**Keywords:** Microcirculation, kidney, CiteSpace, bibliometrics, web of science database

## Abstract

**Background:**

Renal microcirculation plays a pivotal role in kidney function by maintaining structural and functional integrity, facilitating oxygen and nutrient delivery, and waste removal. However, a thorough bibliometric analysis in this area remains lacking. Therefore, we aim to provide valuable insights through a bibliometric analysis of renal microcirculation literature using the Web of Science database.

**Methods:**

We collected renal microcirculation-related publications from the Web of Science database from January 01, 1990, to December 31, 2022. The co-authorship of authors, organizations, and countries/regions was analyzed with VOSviewer1.6.18. The co-occurrence of keywords and co-cited references were analyzed using CiteSpace6.1.R6 software to generate visualization maps. Additionally, burst detection was applied to keywords and cited references to forecast research hotspots and future trends.

**Results:**

Our search yielded 7462 publications, with the American Journal of Physiology-Renal Physiology contributing the most articles. The United States, Mayo Clinic, and Lerman Lilach O emerged with the highest publication count, indicating their active collaborations. ‘Type 2 diabetes’ was the most significant keyword cluster, and ‘diabetic kidney disease’ was the largest cluster of cited references. ‘Cardiovascular outcome’ and ‘diabetic kidney diseases’ were identified as keywords in their burst period over the past three years.

**Conclusion:**

Our bibliometric analysis illuminates the contours of nephrology and microcirculation research, revealing a landscape ripe for challenges and the seeds of future scientific innovation. While the trends discerned from the literature emerging opportunities in diagnostic innovation, renal microcirculation research, and precision medicine interventions, their translation to clinical practice is anticipated to be a deliberate process.

## Introduction

The kidney is a vital organ responsible for maintaining multiple aspects of homeostasis in the human body. Adequate oxygen delivery and utilization at the microcirculatory and cellular levels are essential for optimal organ function. Despite their small size, both kidneys receive a significant proportion of cardiac output, accounting for approximately 20% ∼ 25% of total blood flow (renal blood flow (RBF) is equivalent to 1–1.2 L/min) in adults. The renal artery supplies blood to the kidney, with a complex and dynamic microcirculatory network responsible for maintaining adequate intraglomerular pressure, peritubular capillary pressure, perfusion, and oxygenation. These factors are fundamental for supporting proper glomerular filtration rate (GFR) and overall renal function [1]. The dynamic nature of renal microcirculatory function is critical in regulating the interstitial and intracapillary forces that govern glomerular filtration, salt and fluid reabsorption, and the regulation of the medullary concentration gradient [2,3].

However, the complex architecture of the renal microvasculature and tubular system, coupled with the high energy demands, make the kidney highly susceptible to ischemic or hypoxic injury [4,5]. Numerous renal diseases, such as acute kidney injury [4], chronic kidney disease [6], and diabetic nephropathy [7], are associated with renal microcirculatory dysfunction. Despite the importance of the renal microcirculation in the pathophysiology of these diseases, limited data are currently available on its functional status during physiological and pathological events.

To improve our understanding of the relationship between renal microcirculation and disease, it is necessary to gain valuable insights and promote development in this field. Therefore, researchers must strive to deepen their understanding of the current status of the renal microcirculation. Bibliometric analysis is a quantitative and qualitative approach to evaluate scientific output and research trends within a specific field of study [8]. To date, no bibliometric analysis has been performed on renal microcirculation.

Our aim is to use this method to investigate the current status and emerging trends in renal microcirculation research. This analysis will provide a comprehensive assessment of the developmental status of this field, which will serve to inform researchers and guide their future work. By identifying the most influential authors, institutions, and countries/regions, as well as the most cited articles and journals, we can gain a better understanding of the current state of research in this field. This information can be used to identify gaps in knowledge, prioritize research areas, and facilitate international collaborations.

## Methods and materials

### Search strategies and data collection

To ensure a multidisciplinary perspective in our bibliometric analysis, we conducted a comprehensive search of the Science Citation Index Expanded (SCIE) for biomedical and scientific literature and the Social Science Citation Index (SSCI) for literature that examines the socio-economic, policy, and interdisciplinary aspects of renal microcirculation in the databases of the Web of Science Core Collection (WoSCC). This inclusive approach allows for a broader understanding of the factors influencing the field beyond the biological sciences. Additionally, to ensure retrieval accuracy, we used topic retrieval and set the search terms to TS = (renal microcirculation) OR (renal microvascular) OR (kidney microcirculation) OR (kidney microvascular). Our search strategy was designed to encompass topic-based retrieval. This approach includes the analysis of abstracts where the search terms are prevalent, ensuring the inclusion of pertinent studies in renal microcirculation.

We limited the search period to January 1, 1990, to December 31, 2022, and selected "article" and "review article" as document types. The choice of the time frame, starting from January 1, 1990, to December 31, 2022, was based on the premise that significant advancements in the understanding of renal microcirculation occurred during the early 1990s due to technological innovations in imaging and molecular analysis (Supplementary Figure 1). These advancements marked a new era in the study of renal microvascular functions and pathophysiology, therefore, allowing to capture the most transformative period in renal microcirculation research. In the current investigation, ‘academic literature’ was demarcated to encompass solely peer-reviewed ‘articles’ and ‘review articles’ that embody the substantive discourse in renal microcirculation research. Notwithstanding the precision of our initial query parameters to the WOS for these document types, we undertook a thorough manual verification of the retrieved records, thereby ensuring that our analysis was predicated on a foundation of peer-reviewed scholarly communication. A total of 7,462 records were retrieved, including 6,086 articles and 1,376 reviews. We downloaded the dataset from WoSCC in plain and tab-delimited text format and exported it as ‘Full Record and Cited References’.

### Data analysis

We organized and presented the data in a tabular format using Microsoft Excel 2019. Publications that met the selection criteria were categorized by year, and a year distribution map was created. We used VOSviewer (version 1.6.16) to analyze collaborations between publications. Co-authorship countries/regions, institutions, and authors were identified and represented by nodes of different sizes based on the number of publications. Nodes belonging to the same cluster were represented by the same color, indicating close collaboration in this area. The thickness of the lines connecting the nodes represented the strength of the collaboration between them. We chose only one unit of analysis (countries/regions, organizations, authors) at a time and set the minimum unit of number of documents examined as 5 and the maximum number of total link strengths as 1000.

We also used CiteSpace (version 6.1.R6) for data analysis. The time slice was from 1990 to 2022 with one year per slice, and reference and keywords were selected as node types. The scale factor k was set to 25 for keyword analysis and 8 for reference analysis due to software limitations. Pruning methods were set to ‘pathfinder’ and ‘pruning sliced networks’, while other parameters were left at their default values. Co-citation and co-occurrence network maps were generated to analyze references and keywords of publications, respectively.

## Results

### Basic situations of publications

**Figure 1.**
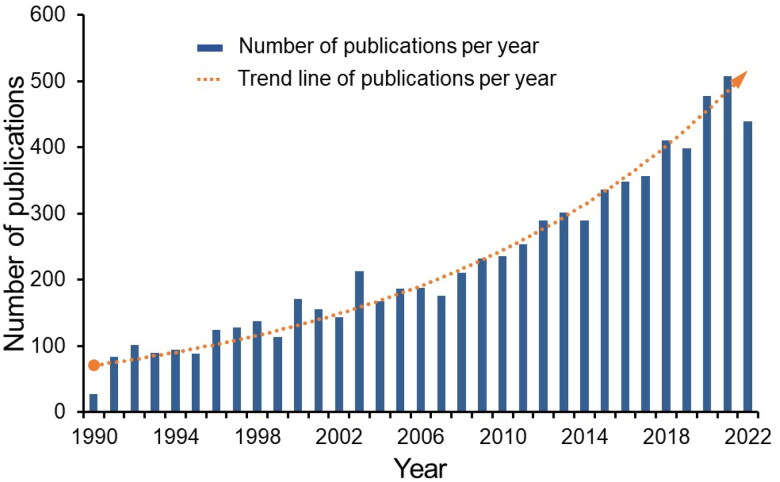
Annual publication trends of renal microcirculation studies in Web of Science from 1990 to 2022. The blue histogram signifies the annual publication volume, while the orange scatter plot illustrates the trend of annual publication volume obtained *via* exponential function fitting.

This study included a comprehensive analysis of 7,462 publications that satisfied the predefined literature criteria. These publications originated from 5,669 institutions across 105 countries/regions and were disseminated through 1,582 distinct journals. The top three journals that published the most articles on this topic were the American Journal of Physiology-Renal Physiology (261 articles), Kidney International (191 articles), and the Journal of the American Society of Nephrology (122 articles), as delineated in Table 1. Figure 1 presents a year-by-year distribution of these publications, demonstrating that research on renal microcirculation has maintained a high level of activity over the past two decades. Notably, a peak was observed in 2021, despite some irregular fluctuations. Given these trends and the inherent characteristics of this evolving field, it can be inferred that, while academic research on renal microcirculation is bustling, it hasn’t yet reached a stage of full maturation. Consequently, there remains a substantial opportunity for future exploration and development within the realm of renal microcirculation studies.

**Table 1.** Top 10 Journals related to renal microcirculation.

Journal	Count	Rank
AMERICAN JOURNAL OF PHYSIOLOGY-RENAL PHYSIOLOGY	261	1
KIDNEY INTERNATIONAL	191	2
JOURNAL OF THE AMERICAN SOCIETY OF NEPHROLOGY	122	3
DIABETES CARE	114	4
HYPERTENSION	113	5
PLOS ONE	111	6
TRANSPLANTATION	96	7
NEPHROLOGY DIALYSIS TRANSPLANTATION	91	8
DIABETOLOGIA	82	9
JOURNAL OF HYPERTENSION	79	10

### Country/region and institutional distribution, and analysis of collaborative relationships

As exhibited in Tables 2 and 3, the United States takes the lead in renal microcirculation-related publications, representing 34.8% of the global output, followed by China (11%), Germany (8.8%), and England (7.5%). These nations have emerged as predominant research contributors in this area. Most of the institutions with the most publications are situated in developed countries/regions, underscoring their substantial contribution to the progress of renal microcirculation research, while also accentuating the disparity between developed and developing nations. The Mayo Clinic (123 publications) and Harvard University (119 publications), both located in the United States, stand out due to their significant influence in this field.

**Table 2.** Top 10 Countries/regions most influential in the field of renal microcirculation in terms of number of publications and total link strength.

Country	Record	Co-authorship country/region	Total link strength	Rank
USA	2596	USA	1487	1
People’s Republic of China	826	England	728	2
Germany	656	Germany	697	3
England	557	Australia	526	4
Italy	495	Canada	520	5
Japan	495	Italy	499	6
Netherlands	381	Netherlands	483	7
Canada	373	France	452	8
France	361	People’s Republic of China	309	9
Australia	346	Sweden	300	10

**Table 3.** The top 10 institutions in the field of renal microcirculation in terms of number of publications and total link strength.

Institution	Record	Co-authorship institution	Total link strength	Rank
Mayo Clinic	123	Melbourne University	328	1
Harvard University	119	Johns Hopkins University	271	2
Washington University	105	Monash University	262	3
Melbourne University	103	Washington University	259	4
Pittsburgh University	102	Michigan University	244	5
Monash university	90	Natl Singapore University	240	6
Toronto University	83	Toronto University	240	7
Johns Hopkins University	75	Sydney University	223	8
Michigan University	75	Harvard University	222	9
Amsterdam University	71	University College London	190	10

Tables also depict the total number of collaborative links, with higher tallies indicating more intensive collaboration between countries/regions or institutions. The United States (1,487 links), England (728 links), and Germany (697 links) are the top three countries/regions in terms of total link strength. Among institutions, the University of Melbourne exhibits the highest total link strength (328), followed by Johns Hopkins University (271), and Monash University (262), indicating their active collaborations within this research area.

**Figure 2.**
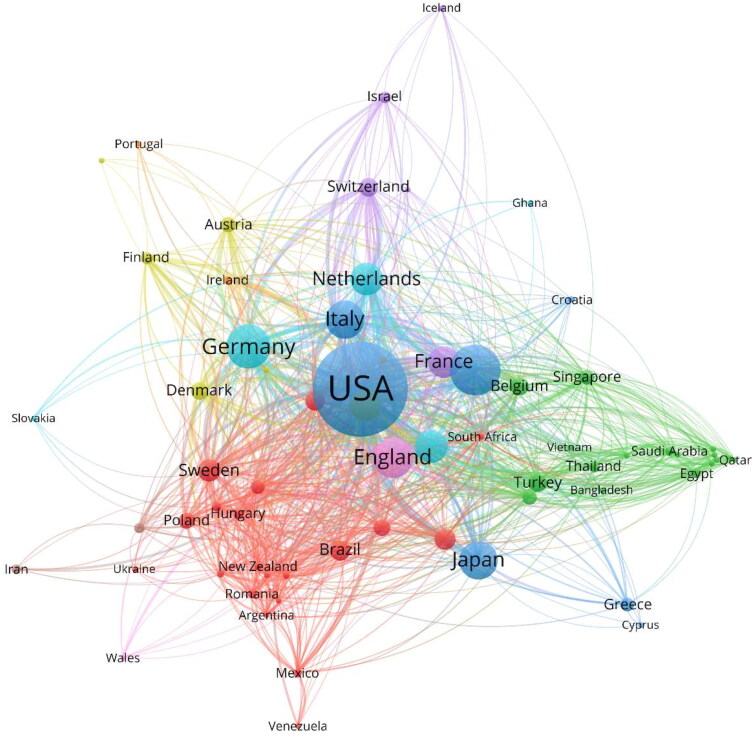
Network visualization map of international collaborations in renal microcirculation research. Nodes represent countries/regions, with their size indicating the volume of publications from each. Colors demarcate different clusters. Links between the nodes denote collaborative relationships, their thickness representing the strength of cooperation.

**Figure 3.**
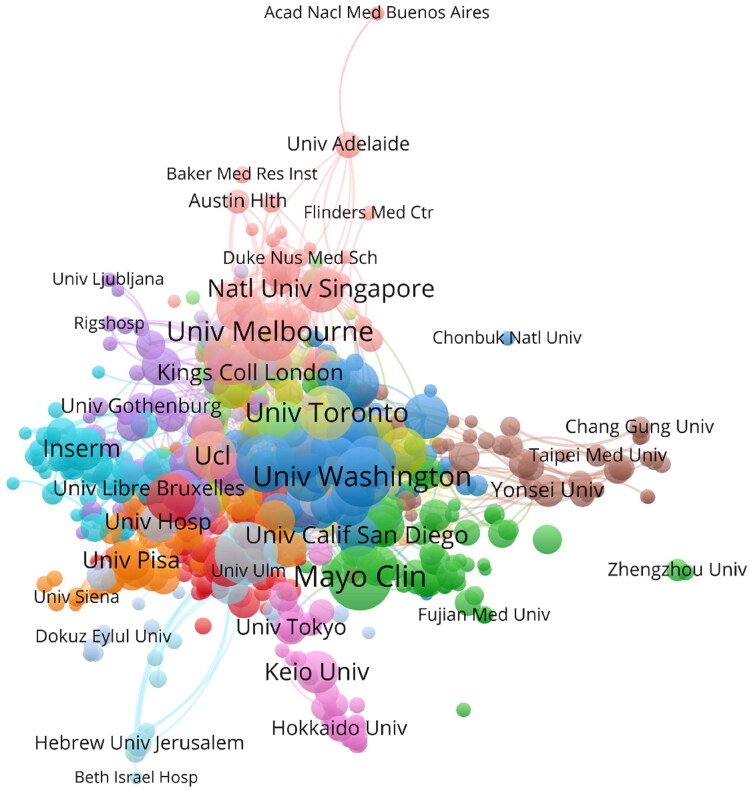
Network visualization map depicting institutional collaborations in renal microcirculation research. Nodes symbolize individual institutions, and their size reflects each institution’s publication volume. Colors designate different clusters. Links between the nodes represent collaborative relationships, with their thickness indicating the strength of cooperation.

Figures 2 and 3 present the national/regional and institutional collaboration maps, respectively, with 67 nodes and 715 nodes. Elements in close collaboration are clustered together. Figure 2 identifies 9 clusters differentiated by color, with red, green, and blue as the primary colors. The red cluster reveals Sweden’s extensive collaboration with countries/regions such as Spain, Scotland, Brazil, Poland, and South Korea. The green cluster showcases Belgium’s fruitful cooperation with countries/regions like Singapore, India, Turkey, Thailand, Saudi Arabia, Malaysia, and Egypt. The blue cluster represents the United States’ collaboration with countries/regions including Italy, China, Japan, Greece, and Croatia.

Figure 3, with its 707 nodes and 18 clusters, shows the University of Melbourne exhibiting the most collaborations with other institutions, such as the University of Sydney, University College London, the University of New South Wales, the University of Queensland, the University of Cambridge, and the National University of Singapore.

### Contributions by authors and their cooperative relationship analysis

**Figure 4.**
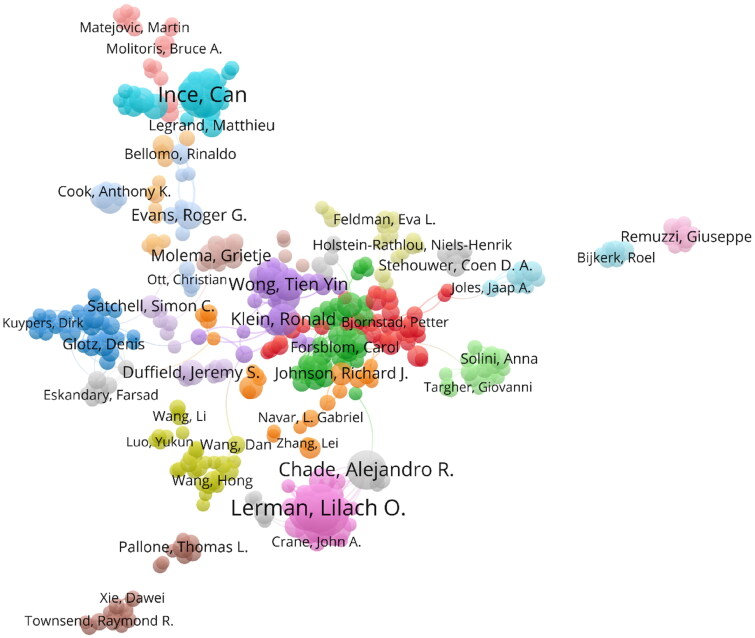
Network visualization map of author collaborations in the field of renal microcirculation. Nodes signify authors, with their size reflecting each author’s publication volume. Colors identify different clusters. Links between nodes illustrate collaborative relationships, with their thickness indicating the strength of cooperation.

This study comprised 3,414 authors, with the ten most prolific listed in Table 4. Lilach O Lerman emerged as the leading scholar, boasting 62 publications. Can Ince (55 articles), Amir Lerman (50), JD Imig (48), and EW Inscho (44) followed next. Lilach O Lerman also demonstrated the most extensive collaborative efforts with a total link strength of 280. Amir Lerman and Xiang-yang Zhu followed with total link strengths of 267 and 186, respectively. The network analysis revealed 385 nodes and 24 clusters (Figure 4), with Lilach O Lerman situated in cluster 8, indicating active collaboration with scholars like Xiang-yang Zhu and Behzad Ebrahimi.

**Table 4.** The top 10 authors in the field of renal microcirculation in terms of number of publications and total link strength.

Author	Records	Co-authorship author	Total link strength	Rank
Lerman Lilach O.	62	Lerman, Lilach O.	280	1
Ince Can	55	Lerman, Amir	267	2
Lerman Amir	50	Zhu, Xiang-yang	186	3
Imig Jd	48	Eirin, Alfonso	169	4
Inscho Ew	44	Textor, Stephen C.	149	5
Chade Alejandro R.	41	Tang, Hui	126	6
Hayashi K	36	Jordan, Kyra l.	112	7
Pallone Tl	34	Zhang, Xin	111	8
Saruta T	33	Ince, Can	105	9
Zhang Xin	18	Ebrahimi, Behzad	90	10

### Analysis of co-occurrence clusters on keywords

**Figure 5.**
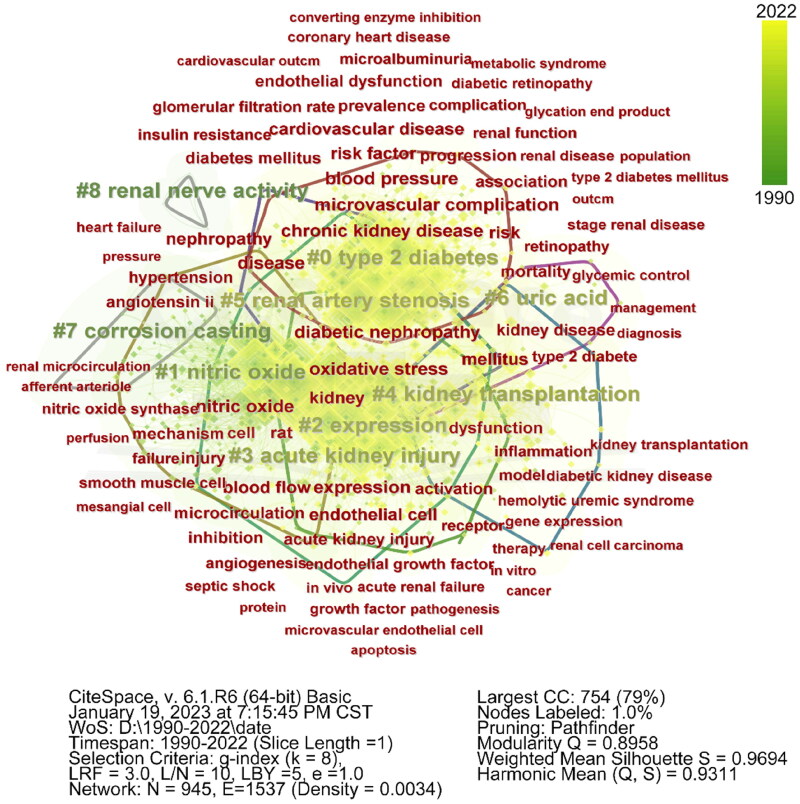
Co-occurrence and clustering analysis of frequently used keywords. Each keyword’s color denotes its frequency, with yellow representing high frequency and blue indicating low frequency.

The analysis of research hotspots is pivotal in identifying a field’s primary focus areas. Keywords epitomize a paper’s core concepts, hence, their visual analysis can reveal research hotspots. Our study consolidated all keywords from 1990 to 2022, merging those with similar meanings. The most frequently occurring keywords were ‘expression’ (605), ‘chronic kidney disease’ (598), and ‘microvascular complication’ (538). We employed CiteSpace6.1.R6 for keyword co-occurrence analysis, which facilitated the co-occurrence and clustering map’s acquisition (Figure 5, Table 5). The network encompassed 5,617 links and 931 nodes with a density of 0.013. The high modularity (*Q* > 0.3) and average silhouette value (*S* > 0.7) indicate distinct cluster boundaries and cluster sizes[9]. The largest cluster (#0) had a silhouette value of 0.789 and majorly explored chronic kidney disease, microvascular complications, and diabetic nephropathy. Other clusters focused on ‘Nitric Oxide’ (#1), ‘Expression’ (#2), and ‘Acute Kidney Injury’ (#3).

**Table 5.** Top 10 Keywords in frequency ranking and their centrality.

Rank	Frequency	Centrality	Keywords
1	605	0.03	expression
2	598	0.02	chronic kidney disease
3	538	0.03	microvascular complication
4	534	0.03	diabetic nephropathy
5	512	0.08	blood pressure
6	490	0.04	oxidative stress
7	489	0.05	nitric oxide
8	483	0.04	disease
9	447	0.02	risk factor
10	434	0.02	nephropathy

### The timeline view of the keywords

**Figure 6.**
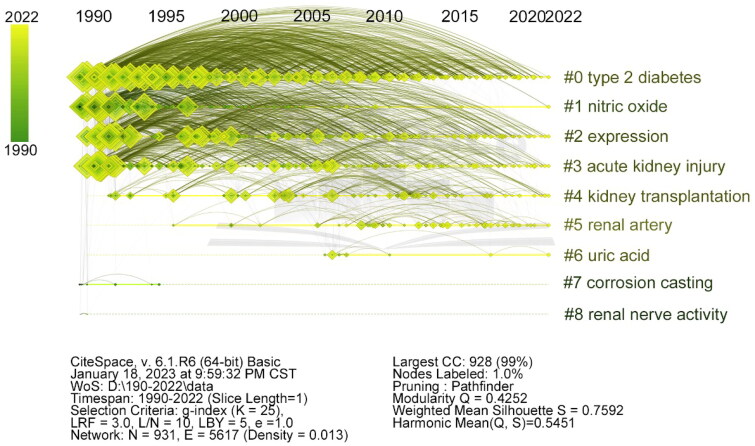
Timeline and clustering view of keywords from selected publications on renal microcirculation. Cluster terms are displayed on the right and arranged in descending order of cluster size. Nodes (papers) are organized horizontally based on their publication year, and links are color-coded according to the year when the link between two nodes (keywords) was first established.

The timeline view arranges each node based on its publication time (horizontal axis) and cluster affiliation (vertical axis). Clusters are displayed in descending order of size. Clusters #0 to #3 have remained active research areas for approximately 30 years. Conversely, clusters #7 and #8, although appearing early, had a lifespan of less than five years (Figure 6).

### Analysis of citation bursts on keywords

**Figure 7.**
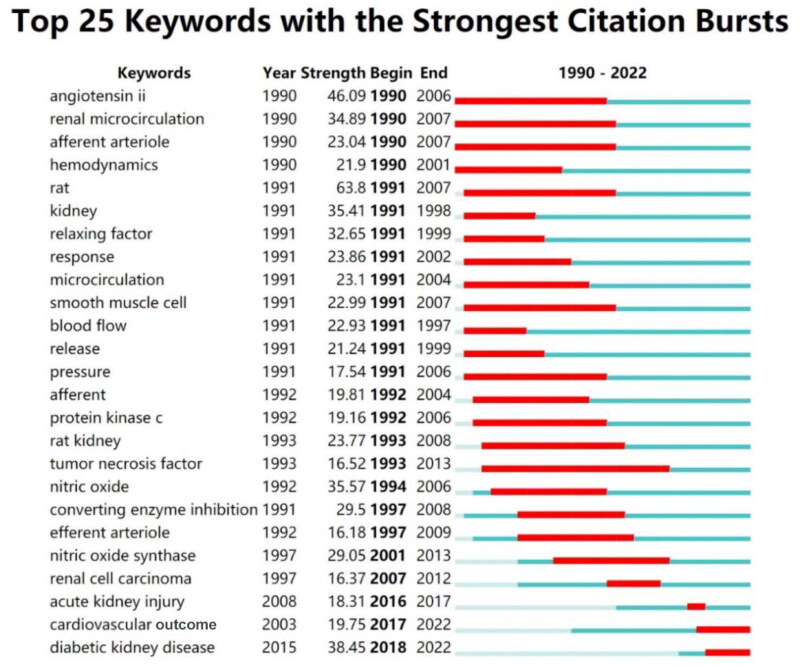
Top 25 keywords with the strongest citation bursts in renal microcirculation research from 1990 to 2022. The red line represents a high frequency of a keyword during the period, while the blue lines denote the time interval.

CiteSpace identified ‘Burst Detection’ after clustering and created a diagram illustrating 25 citation bursts on specific keywords from 1990 to 2022 (Figure 7). The earliest keywords to gain scholarly attention in this field were ‘angiotensin II’, ‘renal microcirculation’, ‘afferent arteriole’, and ‘hemodynamics’. Among these, ‘angiotensin II’ had the highest historical burst strength of 46.09. ‘Tumor necrosis factor’ had the most prolonged burst (1993–2013). Recently, ‘cardiovascular outcome’ and ‘diabetic kidney disease’ emerged as research hotspots with ongoing bursts.

### Analysis of co-cited references

**Figure 8.**
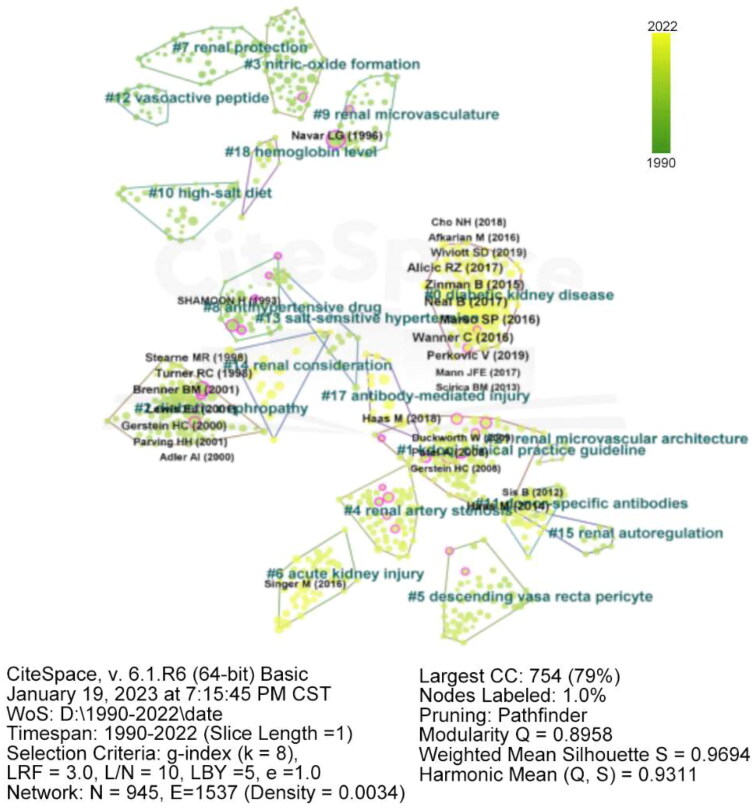
Density visualization map of co-cited references in renal microcirculation research.

**Figure 9.**
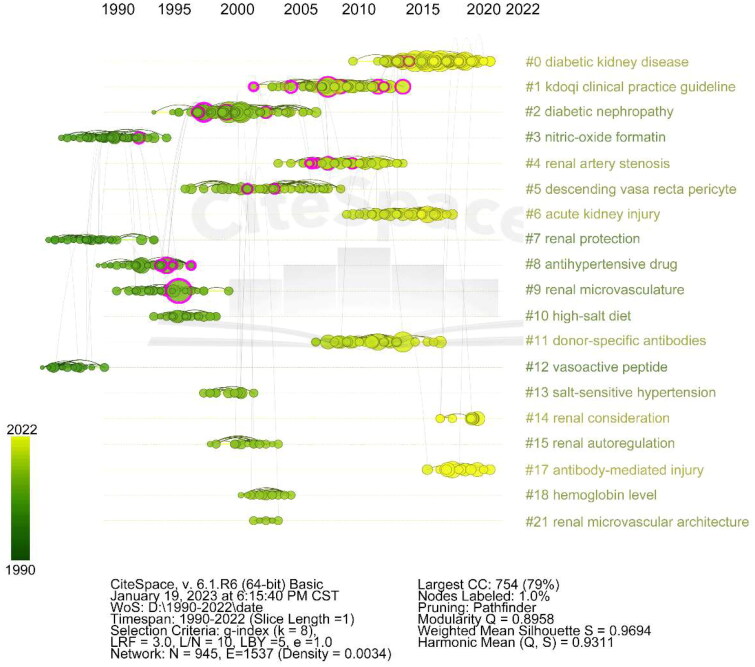
Timeline and clustering view of all co-cited references in renal microcirculation research. Cluster terms are displayed on the right and arranged in descending order of cluster size. Nodes (papers) are organized horizontally based on their publication year, and links are color-coded according to the year when the link between two nodes (references) was first established.

The co-citation analysis conducted using CiteSpace identified the most frequently cited articles in the field (Table 6). The network map (Figures 8 and 9) contains 945 nodes and 1,537 links, with a density of 0.0034. The map comprises 21 clusters, with the largest one (#0) labeled as ‘diabetic kidney disease’ by LLR. This cluster contained 111 members and a silhouette value of 0.991. Cluster #1 was the second largest, with 82 members and a silhouette value of 0.955, labeled as the KDOQI Clinical Practice Guideline. The timeline view of this cluster showcases its temporal evolution. Bright yellow clusters indicate recent research hotspots. Clusters #3, #7, and #12 were initially prominent in the 1990s but experienced a significant research interest decline. Clusters #0, #14, and #17 currently represent the main focus of renal microcirculation research.

**Table 6.** Top 5 co-cited references in frequency ranking and their centrality.

Frequency	Rank	Centrality	Cited References	Tittle
99	1	0.04	Neal B, 2017, NEW ENGL J MED, V377, P644, DOI 10.1056/NEJMoa1611925	Canagliflozin and Cardiovascular and Renal Events in Type 2 Diabetes
87	2	0.02	Marso SP, 2016, NEW ENGL J MED, V375, P311, DOI 10.1056/NEJMoa1603827	Liraglutide and Cardiovascular Outcomes in Type 2 Diabetes
81	3	0.02	Wanner C, 2016, NEW ENGL J MED, V375, P323, DOI 10.1056/NEJMc1611290	Empagliflozin and Progression of Kidney Disease in Type 2 Diabetes
74	4	0.02	Zinman B, 2015, NEW ENGL J MED, V373, P2117, DOI 10.1056/NEJMoa1504720	Empagliflozin, Cardiovascular Outcomes, and Mortality in Type 2 Diabetes
73	5	0.02	Alicic RZ, 2017, CLIN J AM SOC NEPHRO, V12, P2032, DOI 10.2215/CJN.11491116	Diabetic Kidney Disease: Challenges, Progress, and Possibilities

### Analyze of citation burst of references

**Figure 10.**
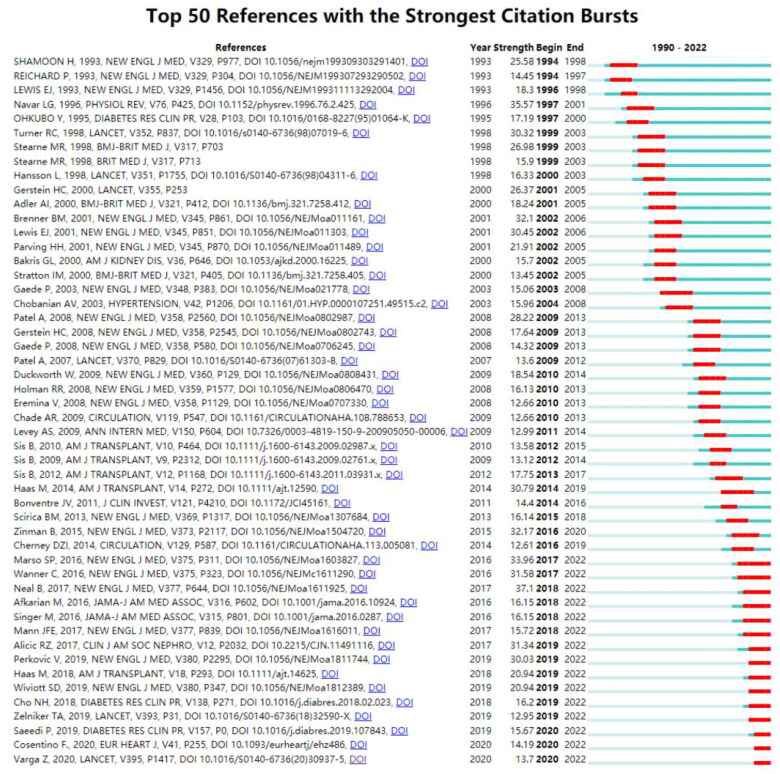
Top 30 references with the strongest citation bursts in renal microcirculation research. The red line represents a high frequency of a reference during the period, while the blue lines denote the time interval.

Burst detection analysis of citation patterns from 1990 to 2022 demonstrated Neal B (2017) in Cluster #0 as the highest-ranked reference, with a burst score of 37.10 (Figure 10). This was followed by Navar LG (1996) in Cluster #9 (burst score of 35.57) and Marso SP (2016) in Cluster #0 (burst score of 33.96). Citation bursts periods were notably short, with the most extended burst period lasting only five years. Interestingly, three references began their citation bursts just three years ago, and their citations are still on the rise, indicating potential recent research hotspots.

## Discussion

The field of nephrology has undergone remarkable developments over recent decades, marked by an enhanced understanding of kidney disease pathophysiology, diagnostics, and treatment modalities. Nonetheless, the landscape of challenges confronting healthcare practitioners and researchers has concurrently evolved, shaped by shifting demographics, the emergence of novel comorbidities, and the advent of new research discoveries. In this study, we employed CiteSpace and VOSviewer to conduct a bibliometric evaluation of renal microcirculation literature published between 1990 and 2022. Our analyses scrutinized not only the spatial and temporal distribution of these publications but also the network of author collaborations, thematic focal points, and the forefront of research in this area.

One of the key metrics for assessing research impact is the volume of publications in peer-reviewed academic journals [10]. Our study elucidates the globally dominant role of the United States in the realm of renal microcirculation research, a dominance that can be ascribed to its technical, economic, and academic prowess. Beyond identifying the most impactful countries/regions in this field, our exploration of the collaboration networks among these countries/regions and researchers has brought to light a key facet of scientific social networks, showcasing the fundamental structures of scientific collaboration. We have revealed a country-level collaboration network. It is evident that countries/regions in the Asian-Pacific region maintain close ties with North American countries/regions, whereas European nations tend to collaborate predominantly within their own region. Notably, European and English-speaking countries/regions exhibit a high degree of international collaboration, a finding that aligns with the publication data retrieved from our database. The top ten institutions in our ranking further underscored the prominent position of the United States. Johns Hopkins University, Washington University, University of Michigan, and Harvard University—all located in the United States—serve to underline the preeminent hierarchy of domestic organizations in terms of collaboration. This observation accentuates the need to augment global collaborations with other countries/regions to diversify scientific contributions and insights.

The elucidation of key terms with pronounced citation bursts, such as ‘expression,’ ‘uric acid,’ and ‘nitric oxide,’ provides the evolving focus of renal microcirculation research. The co-occurrence map facilitates a dichotomous classification of these terms into pathophysiological mechanisms and clinical manifestations. The pathophysiological domain, underscored by the recurrent keyword ‘expression,’ touches on the cellular and molecular dynamics such as endothelial cell function, hemorheological changes, and neural control of the renal microvasculature. Specifically, the influence of Angiotensin II (AngII) on renal microvascular architecture, highlighted by its citation burst, underscores its critical role in modulating microcirculatory dynamics and kidney function [11]. The vascular endothelial-AT1 axis, identified as a stimulant for microvessel growth, unveils potential therapeutic targets. Inhibition of AT1 receptors could modulate post-glomerular microvessel morphology, thereby impacting parameters such as length, volume, surface area, and intercapillary distance, with subsequent effects on kidney perfusion and function. In clinical practice, these insights suggest that Angiotensin II receptor type 1 (AT1) antagonists might be leveraged to alter renal microvascular architecture, potentially offering a novel approach to manage chronic kidney disease progression. Furthermore, AngII's action on arteriolar tone implicates its role in the regulation of glomerular blood flow, with implications for the treatment of conditions characterized by altered renal perfusion [12,13]. Besides, the role of nitric oxide (NO) in renal microcirculation, forming the basis of the second-largest cluster, shifts the focus from vasoconstriction to vasodilation, highlighting NO's counteractive properties against vasoconstrictors like AngII. The therapeutic potential of NO donors or agents that enhance endogenous NO production could be significant, particularly in conditions such as renal ischemia-reperfusion injury. By improving renal medullary perfusion and overall microcirculation, NO-related therapies offer a promising avenue to mitigate renal ischemic damage and improve outcomes in acute kidney injury [14,15]. The integration of key terms with clinical practice underscores a translational research trajectory wherein molecular insights into renal microcirculation could inform the development of innovative therapeutic strategies, such strategies would aim to modify microvascular function and architecture, ultimately improving the management of renal pathologies and enhancing patient care.

The citation bursts observed for keywords reflect the shifting paradigms in nephrology where the intricacies of renal microcirculation are increasingly linked with systemic pathologies. To elucidate, the sustained emphasis on "Ang II" over the years resonates with its established role in pathophysiological states, particularly its impact on renal hemodynamics and blood pressure regulation. This historical focus has paved the way for current therapeutic interventions, such as the use of renin-angiotensin system blockers which have become cornerstone treatments for chronic kidney disease. The enduring investigation into "hemodynamics" underpins this further by accentuating the importance of understanding renal blood flow regulation in kidney health and diseases. In a dynamic temporal perspective, the more recent ascension of "cardiovascular outcome" as a keyword with ongoing bursts mirrors the evolution of nephrology research from a kidney-centric view to a more integrated approach considering the kidney’s relationship with cardiovascular health. This is clinically significant as it directs research efforts toward identifying renal microvascular changes that may precede and predict cardiovascular events, thus creating opportunities for early intervention in at-risk populations. Furthermore, the emergence of "diabetic kidney disease" highlights the critical intersection of metabolic and renal disorders, underscoring the need for strategies that address microvascular damage within the diabetic milieu. This has direct implications for the development of targeted therapies that aim to preserve microvascular integrity and function in the face of hyperglycemic injury, a major contributor to the progression of diabetic nephropathy. The convergence of these keywords points toward an integrative approach in nephrology that aligns molecular and clinical research with public health priorities.

The timeline and clustering of keywords, as presented in Figure 6, reveal a trend that mirrors the scientific community’s growing recognition of renal microcirculation not as an isolated phenomenon but as a pivotal factor in the continuum of renal diseases and their comorbid conditions. The early clusters, representing longstanding areas of investigation, such as the regulation of renal blood flow and the role of Ang II, have laid a robust foundation for understanding the pathophysiology of renal disorders. Building on this, more recent clusters reflect a nuanced appreciation for the interconnectedness between renal microcirculation and systemic health, particularly cardiovascular outcomes and diabetic kidney disease. This shift acknowledges the kidney’s integral role in overall homeostasis and the implications of microvascular dysfunction beyond the organ itself. The temporal progression of these keywords indicates a maturing field that is increasingly focused on the translational potential of basic research, striving for therapeutic interventions that are grounded in a comprehensive understanding of renal and systemic physiology. This progression underscores the necessity for a research framework that not only delves into molecular mechanisms but also addresses the translational leap to clinical application. For instance, the interplay between renal microcirculation disturbances and cardiovascular events highlights the potential for early diagnostic and therapeutic strategies, which could preemptively mitigate the progression of both renal and cardiovascular diseases. Similarly, insights into microvascular damage in diabetes advocate for precision medicine approaches that target the unique pathological processes of diabetic kidney disease.

Our bibliometric analysis reveals emergent trends in research that have the potential to significantly influence clinical practice. Renal microvascular dysfunction is a central pathological feature in the spectrum of nephropathies, with particular relevance to acute kidney injury (AKI) and chronic kidney disease (CKD). These conditions, both linked with dismal prognoses, are intricately associated with the integrity of the renal microcirculatory system [16–19]. The role of microvascular damage in AKI is well-established, with the PI3K/Akt/eNOS signaling axis emerging as a vital mediator in the restoration of renal microvascular homeostasis following injury [20–22]. CKD, often progressing through a fibrotic trajectory, shares a commonality with AKI in its association with microvascular pathology. The therapeutic impact on renal microvascular endpoints has become a focal point of research, particularly within the scope of diabetes management. Agents such as Liraglutide [23] and Empagliflozin [24] have shown to attenuate renal microvascular complications and reduce the risk of renal adverse outcomes, respectively. Canagliflozin, another Sodium-glucose cotransporter 2 (SGLT2) inhibitor, has been demonstrated to decrease the risk of renal failure progression by ameliorating intraglomerular hypertension, offering a protective stance for renal microvasculature [25,26]. Additionally, potential therapeutic strategies targeting renal microcirculation by vasodilators [27], antioxidants [28], anti-inflammatory agents [29], and RAAS inhibitors [30], therefore, has been shown promising results in preclinical and early-phase clinical trials. The integration of renal microcirculatory-targeted strategies insight into clinical practice underscores the need for a translational research trajectory, fostering therapeutic strategies that aim to modify microvascular function and architecture for improved patient outcomes. This is not merely a theoretical proposition; rather, it is a call to action for clinical trials to evaluate the efficacy of interventions.

One limitation of the present study is the inclusion of self-citations in the data analysis. The question of whether to exclude self-citations in scientometric analysis is complex and multifaceted. While exclusion could address issues of citation inflation and bias, it could also inadvertently obscure the developmental nature of research and unfairly disadvantage certain fields or methodologies. A nuanced approach may be required, one that acknowledges the legitimate role of self-citations in academia while remaining vigilant against practices that could distort the scholarly record, considering both the integrity of scientometric indicators and the dynamic character of scientific knowledge production.

In conclusion, our bibliometric analysis underscores a dynamic phase in nephrology and microcirculation research, marked by considerable challenges and the potential for scientific breakthroughs after 2022. We recognize that while the identified trends point to innovative opportunities—such as advances in diagnostic tools, renal microcirculation insights, precision medicine, and telemedicine applications—the direct impact on clinical practice may be non-immediate. These findings should, therefore, be interpreted as signposts directing the research community toward promising areas that may yield tangible clinical benefits following thorough investigation and validation.

## Supplementary Material

Supplemental Material

## Data Availability

The data that support the findings of this study are available from the corresponding author upon reasonable request.
