# Supplementary material for: Tiny clue reveals the general trend: a bibliometric and visualized analysis of renal microcirculation
Source: Ren Fail. 2024 Mar 14;46(1):2329249. doi: 10.1080/0886022X.2024.2329249 (PMC10946277; doi:10.1080/0886022X.2024.2329249)
Supplement: Supplemental Material [file IRNF_A_2329249_SM5895.pdf]

Supplementary Information

Tiny clue reveals the general trend: a bibliometric and visualized analysis of renal microcirculation

Running Head: A bibliometric and visualized analysis of renal microcirculation

*Bing Wang12#, Mengting Xu12#, Sunjing Fu12, Yingyu Wang12, Hao Ling3, Yuan Li12, Bingwei Li12, Xueting Liu12, Qin Ouyang4, Xiaoyan Zhang12, Ailing Li12, Xu Zhang5, Mingming Liu126**

[1] Institute of Microcirculation, Chinese Academy of Medical Sciences & Peking Union Medical College, Beijing 100005, China

[2] International Center of Microvascular Medicine, Chinese Academy of Medical Sciences, Beijing 100005, China

[3] Department of Radiology, The Affiliated Changsha Central Hospital, Hengyang Medical School, University of South China, Changsha 410004, China

[4] Department of Pathology, Wangjing Hospital, China Academy of Chinese Medical Science, Beijing 100102, China

[5] Laboratory of Electron Microscopy, Ultrastructural Pathology Center, Peking University First Hospital, Beijing, 100034, China

[6] Diabetes Research Center, Chinese Academy of Medical Sciences & Peking Union Medical College, Beijing 100005, China

[#] These authors contributed equally to this study. * Corresponding authors.

*Corresponding authors to whom page proofs and reprint requests should be addressed: Mingming Liu, Institute of Microcirculation, Chinese Academy of Medical Sciences & Peking Union Medical College (CAMS & PUMC), No.5 Dong Dan Third Alley, Dongcheng District, Beijing, 100005, China. (E-mail: mingmingliu@imc.pumc.edu.cn) ORCID: 0000-0002-6750-5068)

Information Classification: General

To enable a robust comparative evaluation of research productivity before and after the year 1990, we have integrated a supplementary figure that illustrates the academic output related to renal microcirculation research from 1970 to 1989, as indexed in the Web of Science. The discernible paucity of annual publications within this timeframe offers empirical justification for designating 1990 as the pivotal year for initiating our search criteria. This temporal demarcation was chosen to reflect the inflection point in scholarly attention, as evidenced by the substantive increase in publication volume post-1990, thereby providing a clear contrast with the prior two decades of modest research activity in this field.

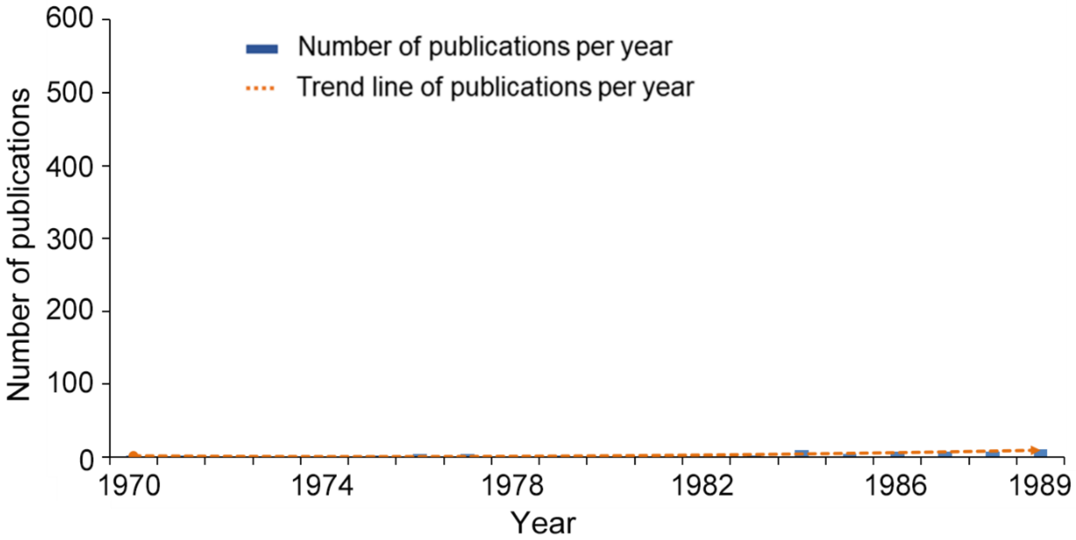

Supplementary Figure 1. Annual publication trends of renal microcirculation studies in Web of Science from 1970 to 1989. The blue histogram signifies the annual publication volume, while the orange scatter plot illustrates the trend of annual publication volume obtained via exponential function fitting.
